# The Effect of Rifle Carriage on the Physiological and Accelerometer Responses During Biathlon Skiing

**DOI:** 10.3389/fspor.2022.813784

**Published:** 2022-03-25

**Authors:** Craig A. Staunton, Luciën Sloof, Maxime Brandts, Malin Jonsson Kårström, Marko S. Laaksonen, Glenn Björklund

**Affiliations:** ^1^Swedish Winter Sports Research Centre, Faculty of Human Sciences, Mid Sweden University, Östersund, Sweden; ^2^Institute of Sports Science, Saarland University, Saarbrücken, Germany

**Keywords:** athlete, IMU, physiology, PlayerLoad™, undulating terrain

## Abstract

**Purpose:**

Investigate the effect of biathlon rifle carriage on physiological and accelerometer-derived responses during biathlon skiing.

**Methods:**

Twenty-eight biathletes (11F, 17M) completed two XC skiing time-trials (~2,300 m), once with and once without the biathlon rifle, with concurrent measurements of HR, skiing speed and accelerations recorded from three triaxial accelerometers attached at the Upper-spine, Lower-spine and Pelvis. Exercise intensity was quantified from HR, skiing speed as well from accelerometry-derived PlayerLoad™ per minute (PL·min^−1^) and average net force (AvF_Net_). All metrics were analyzed during Uphill, Flat and Downhill sections of the course. Relationships between accelerometry-derived metrics and skiing speed were examined.

**Results:**

Time-trials were faster for males compared with females (mean difference: 97 ± 73 s) and No-Rifle compared to With-Rifle (mean difference: 16 ± 9 s). HR was greatest during Downhill (183 ± 5 bpm), followed by Uphill (181 ± 5 bpm) and was lowest in the Flat sections (177 ± 6 bpm, p <0.05). For PL·min^−1^ and AvF_Net_ there were 3-way Rifle x Gradient x Sensor-Position interactions. Typically, these metrics were greatest during Uphill and Flat sections and were lowest during Downhill sections. Rifle carriage had no impact on the AvF_Net_ at the Lower-Spine or Pelvis. Significant positive linear relationships were identified between skiing speed and accelerometer-derived metrics during Uphill, Flat and Downhill skiing (*r* = 0.12–0.61, *p* < 0.05).

**Conclusions:**

The accelerometry-derived approach used in this study provides the potential of a novel method of monitoring the external demands during skiing. In particular, AvF_Net_ with sensors located close to the center of mass displayed greatest utility because it followed the expected response of external intensity where responses were greatest during uphill sections, followed by flats and lowest during downhills. In addition, there were significant positive relationships between AvF_Net_ and skiing speed ranging from small to large. Accelerometry-derived measures could provide useful estimates of the external demands in XC skiing and biathlon.

## Introduction

Biathlon is an Olympic winter sport which combines cross-country (XC) skiing using the skating technique with precision shooting. Performance in biathlon is determined by a mixture of skiing speed, shooting accuracy and shooting time (Laaksonen et al., 2018). It has been suggested that the skiing component of biathlon can explain 50–65% of the variation in overall performance, depending on the specific biathlon event (Luchsinger et al., 2018, 2019, 2020).

During the skiing component of biathlon competitions, athletes are required to carry a rifle (minimum mass: 3.5 kg) harnessed on their back. Despite this, it has been reported that even top-level biathletes complete surprisingly little (15–20%) of their endurance training actually carrying the rifle (Laaksonen et al., 2018). Regardless, several researchers have observed that the rifle carriage increases the physiological responses (i.e., energy cost, heart rate (HR) and blood lactate) whilst roller-skiing on a treadmill (Rundell and Szmedra, 1998; Stöggl et al., 2015; Jonsson Kårström et al., 2019). However, limited research has observed the impact of rifle carriage on physiological responses in actual on-snow conditions. It is useful for coaches and athletes to understand how rifle carriage impacts physiological response to XC skiing during biathlon competitions and training sessions.

It is common for coaches and athletes in biathlon (and XC skiing) to utilize HR as a means of prescribing and monitoring the internal responses to exercise (Tønnessen et al., 2014). The basis of HR as a measure of exercise intensity is rooted in the assumption of a linear relationship between HR and $\overset{∙}{\text{V}}{\text{O}}_{2}$ during steady-state sub-maximal intensity exercise (Hopkins, 1991), where research has shown nearly perfect correlation coefficients (*r* = 0.99) (Swain et al., 1998; Schrack et al., 2014). However, previous studies have demonstrated that skiers are exposed to supramaximal intensity efforts during the uphill sections of a XC skiing course (i.e., contribution from anaerobic energy sources) (Karlsson et al., 2018; Gløersen et al., 2020). Further, other studies have demonstrated that XC skiers employ pacing strategies during competitions which means that the metabolic intensity during biathlon and XC skiing is intermittent (Björklund et al., 2011; Andersson et al., 2016). Therefore, the utility of HR as a measure of exercise intensity in XC skiing and biathlon is questionable.

Wearable accelerometers offer a measurement system to quantify the external demands of sports in a manner which can overcome the limitations of HR. This is because accelerometers have a sufficient measurement resolution to reflect instantaneous changes in exercise intensity during intermittent activity (Boyd et al., 2011; Staunton et al., 2017). In addition, wearable accelerometers offer a measurement system which might be able to quantify the biomechanical intensity of activity in addition with the locomotor demands (Boyd et al., 2011; Vanrenterghem et al., 2017). Accordingly, accelerometry-derived metrics, such as PlayerLoad™ (PL) and average net force (AvF_Net_), have readily been used for athlete monitoring in team sports, such as basketball (Staunton et al., 2017, 2018a), soccer (Scott et al., 2013; Dalen et al., 2016), and Australian football (Cormack et al., 2013; Mooney et al., 2013).

One of the major contributors to accelerometry-derived measures of exercise are the vertical ground reaction forces experienced during foot strikes whilst running (Boyd et al., 2011). As a consequence, it is known that very strong relationships exist between running speed and both PL (Barrett et al., 2014) and AvF_Net_ (Staunton et al., 2017) during over ground running. However, accelerometry-derived metrics, such as PL and AvF_Net_, have not been utilized to monitor the external demands during any form of on-snow skiing. Therefore, the relationships between accelerometer-derived metrics with locomotion speed during on-snow skiing remain unknown, despite the potential implications of these relationships on the utility of accelerometry for monitoring the external demands during skiing.

Therefore, the aims of this study were to investigate: (1) the impact of rifle carriage on the physiological responses to the XC skiing component of biathlon during on-snow conditions; and (2) the utility of accelerometry as a means to measure the external demand of the XC skiing component of biathlon, including investigating relationships with skiing speed.

## Methods

### Participants

In accordance with the primary aim of this study, an a-priori power calculation was conducted in G^*^Power using a repeated measured ANOVA within factors (gradient: three levels) model. It was determined that 28 participants were needed to provide 80% power at an alpha value of 0.05 assuming a true effect size and correlation among repeated measures that was moderate (*f* = 0.25; *r* = 0.5) and a non-sphericity correction equal to 1. Accordingly, 28 biathletes (11 females: Age: 19 ± 2 years, Stature: 167 ± 7 cm, Body mass: 68 ± 10 kg; 17 males: Age: 19 ± 2 years; Stature: 181 ± 5 cm; Body mass: 74 ± 6 kg) who regularly compete in biathlon were recruited to participate in this study. The biathletes were developmental tier 2 athletes according to participant classification framework (McKay et al., 2022). All participants provided written informed consent and completed all requirements of the study. The regional ethical review board in Umeå, Sweden (registration number: 2016-506-31M) preapproved the research techniques and experimental protocol. All research was conducted in accordance with the Code of Ethics of the World Medical Association (Declaration of Helsinki).

### Design

**Figure 1:**
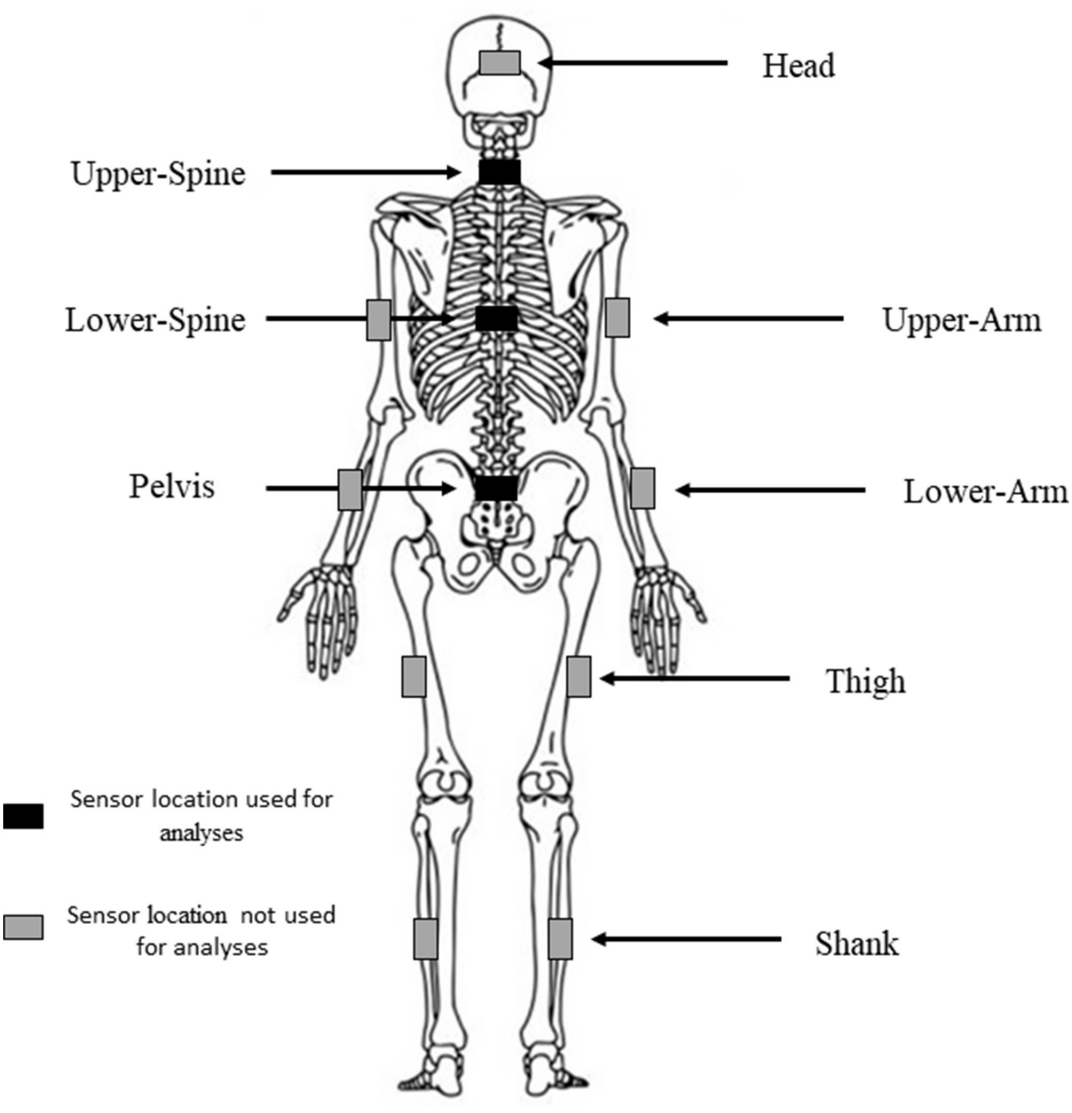
Depiction of the IMU sensor locations during the XC skiing time-trials.

Participants completed two maximal effort XC skiing time-trials, once with and once without carrying the biathlon rifle in a randomized-counterbalanced order. During the time-trials, athletes wore the MyoMotion (Noraxon Inc., Scottsdale, AZ, USA) 3D motion analysis system which consisted of 12 inertial measurement units (IMU) attached to various points of the body. The MyoMotion system contains inertial sensors which provides information of bodily movements recording at 100 Hz (technical specifications: accelerometer: ± 16 g; gyroscrope: ± 2,000°·s^−1^; magnetometer: ±1.9 gauss). The MyoMotion system has confirmed validity and reliability (Yoon, 2017; Yoon et al., 2019) for measuring three-dimensional angular movements. Accelerations derived from three of the 12 IMUs were used for analysis in this study. Those were the three IMUs positioned along the spine and included the Pelvis, Lower-Spine and the Upper-Spine positions (Figure 1). These positions were chosen because they are commonly used attachment points for measuring exercise volume and intensity in sports (Barrett et al., 2014; Staunton et al., 2017). Additionally, skiing speed was calculated from the change in position per time (Forerunner 920; Garmin, Olathe, KS, USA), HR (Polar Electro OY, Kempele, Finland) was recorded throughout the time-trials and blood lactate (Bla) was recorded immediately prior to- and 2-min post time-trial.

### Skiing Course

**Figure 2:**
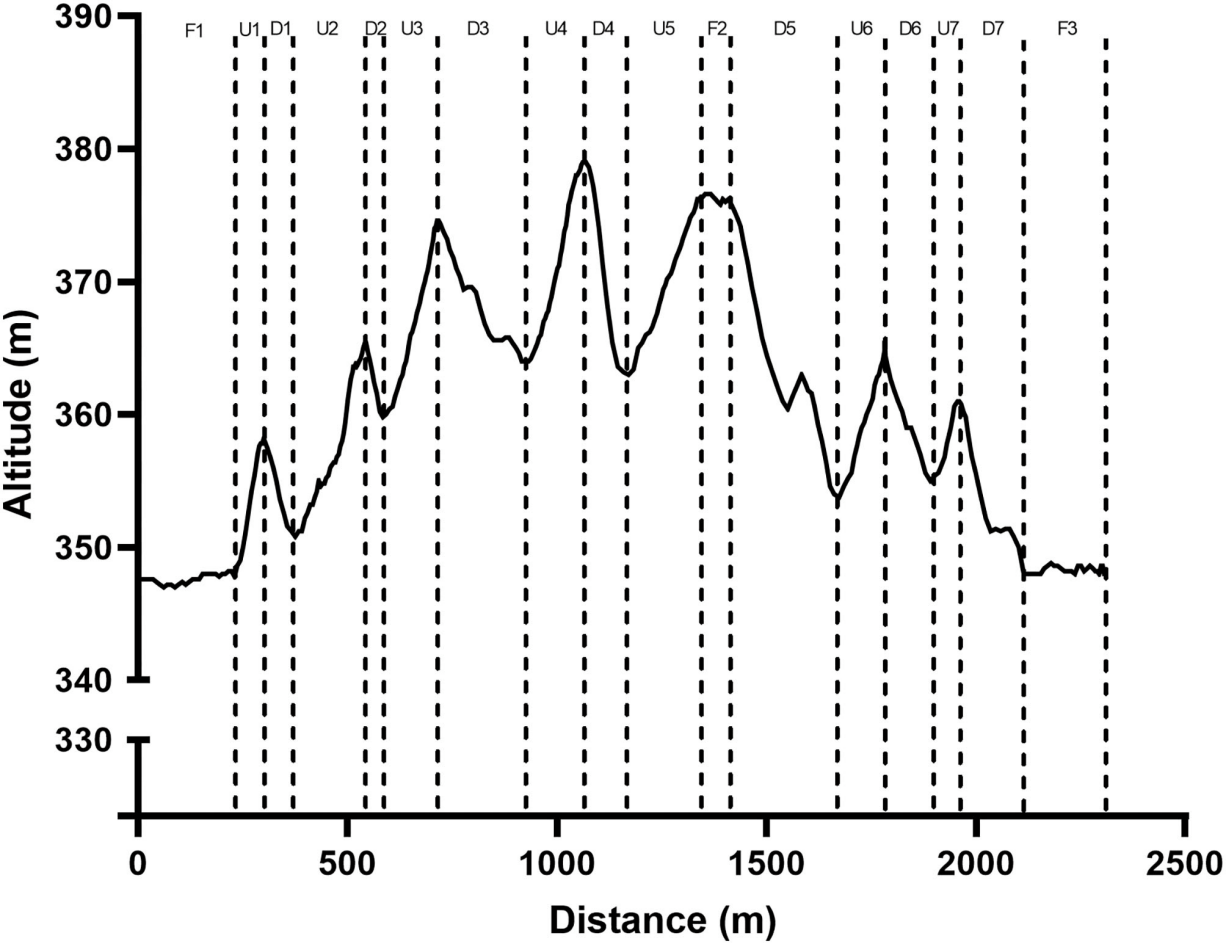
Course profile including Uphill, Flat and Downhill sections. U, Uphill; F, Flat; D, Downhill.

Prior to commencement of the time-trials, athletes completed a standardized 15-min warm-up which comprised of familiarization of the time-trial loop and dynamic bodily movements. The time-trials were performed at the Swedish National Biathlon Arena on a race circuit which is regularly included in the IBU world cup event. The circuit was ~2,300 m in distance with a total climb of 95 m. Participants competed at least 20-min of self-regulated active recovery between time-trial efforts, which previous research has demonstrated is sufficient for blood lactate clearance (Vesterinen et al., 2009; Menzies et al., 2010). The course was divided into discrete uphill (U1, U2, U3, U4, U5, U6, U7), downhill (D1, D2, D3, D4, D5, D6, D7), and flat sections (F1, F2, F3) for analyses by visual inspection of the altitude profile from the positioning system worn by athletes (Figure 2).

### Data Analyses

Accelerometer data from the Pelvis, Lower-Spine and Upper-Spine IMUs were downloaded using the manufacturer software (MyoResearch v3.6.2; Noraxon Inc., Scottsdale, AZ, USA). MyoMotion acceleration data were continuously transformed from sensor to world coordinates by applying a sensor fusion algorithm from the onboard gyroscopes and magnetometers. Accordingly, 1 g was subtracted from the vertical axis in order to remove the gravitational component from the acceleration signal. Following this, a low-pass 4th order Butterworth filter with a cut-off frequency of 15 Hz was used to remove the noise component of the signal. This frequency was identified from visual inspection of the energy spectrum of the acceleration signal and is closely aligned with previous research which has used similar low-pass filter frequencies during sports (Wundersitz et al., 2015a,b).

Exercise intensity was quantified from the accelerometer signals for each section of the skiing course and in each sensor position in two ways. Firstly, PlayerLoad™ per minute (PL·min^−1^) was calculated as previously described (Boyd et al., 2011). Briefly, PL is calculated as the square root of the sum of the squared instantaneous rate of change in acceleration in each of the three axes (X, Y, and Z axis) and divided by 100. Subsequently, the accumulated PL for each section of the skiing course was divided by the time taken to calculate PL·min^−1^.

Secondly, accelerometry-derived average net force (AvF_Net_) was calculated as previously described (Equation 1) (Staunton et al., 2017, 2018a,b, 2020). Briefly, the product of the filtered instantaneous resultant acceleration vector and participant's body mass was used to determine instantaneous net force (F_Net_), which was then averaged over user selected periods. This metric has confirmed construct validity to measure exercise intensity in basketball (Staunton et al., 2017) and is strongly correlated with $\overset{∙}{\text{V}}{\text{O}}_{2}$ and running speed on flat surfaces (Staunton et al., 2017, 2018a). The rifle mass was not included in the AvF_Net_ calculation during the with-rifle trial in order to determine the impact of the rifle on the accelerometer response.


(1) $$\begin{array}{r} AvF_{Net}=BM\;\times \;\frac{\sum_{i=1}^{n}\left(\sqrt{a_{x_{i}}^{2}+a_{y_{i}}^{2}+a_{z_{i}}^{2}}\right)}{n} \end{array}$$

Equation 1: Accelerometry-derived average net force.

*AvF*_*Net*_ = *Average net force, BM* = *body mass, a*_*x*_ = *acceleration in the x direction, a*_*y*_ = *acceleration in the y direction, a*_*z*_ = *acceleration in the z direction, n* = *number of samples*.

In addition, mean HR (% maximum activity) and skiing speed (m·s^−1^) were calculated for each section of the skiing track. Blood lactate (Bla) was recorded approximately 2-min prior to the beginning of each time-trial and approximately 2-min following completion of each time-trial using an automated analyzer (Biosen S-line; EKF diagnostics, Magdeburg, Germany).

For the purposes of comparing uphill, downhill and flat sections, all metrics from the uphill sections (U1, U2, U3, U4, U5, U6, U7) were averaged and considered as Uphill; all metrics from the downhill sections (D1, D2, D3, D4, D5, D6, D7) were averaged and considered as Downhill; and all flat sections (F1, F2, F3) were averaged and considered as Flat.

### Statistical Analyses

Statistical analyses were performed using IBM SPSS Statistics (Version 27.0; IBM Corporation, NY) with level of significance set at α <0.05. Shapiro-Wilk tests confirmed that the assumption of normality was not violated and group data were expressed as mean ± standard deviation (SD). A two-way mixed-model analysis of variance (ANOVA) was used to determine the effect of Sex (Between Factors: Female; Male) and/or Rifle (Within Factors: With-Rifle; No-Rifle) on time-trial performance. Repeated measures two-way ANOVAs (within factors: Gradient; Rifle) were used to identify if Gradient (Uphill; Downhill; Flat) and/or Rifle (With-Rifle; No-Rifle) influenced the response of HR throughout the time-trial. Repeated measures two-way ANOVAs (within factors: Timing; Rifle) were used to identify if Timing (Baseline; Pre; Post) and/or Rifle (With-Rifle; No-Rifle) influenced the response of Bla to the time-trial. Repeated measures three-way ANOVAs (within factors: Gradient; Rifle; Sensor Position) were used to identify if Gradient (Uphill; Downhill; Flat); Rifle (With-Rifle; No-Rifle) and/or Sensor Position (Upper-Spine; Lower-Spine; Pelvis) influenced the response of accelerometry-derived metrics (PL·min^−1^; AvF_Net_) throughout the time-trial. Significant interactions were followed up with simple main effect analyses with pairwise comparisons using Bonferroni correction. Furthermore, Pearson correlation coefficients (*r*) were used to examine the strength of relationship between skiing speed and accelerometer-derived metrics during Uphill, Downhill and Flat skiing sections at all sensor locations. To compare the strength of relationship between skiing speed and accelerometer-derived metrics at all sensor locations, the Pearson correlation coefficient r values were z-transformed using Fisher's z-transformation (Fisher, 1915). This analysis identified that there was minimal differences in the strength of relationship between sensor locations. Accordingly, for simplicity, only the relationships obtained at the Pelvis sensor location are reported because this is considered as the criterion sensor location due to being closest to the center of mass (Halsey et al., 2011). Strength of relationships were evaluated according to methods previously stated (Hopkins, 2002). Very small correlations were 0.0–0.1; small correlations were 0.1–0.3; moderate correlations were 0.3–0.5; large correlations were 0.5–0.7; very large correlations were 0.7–0.9 and nearly perfect correlations were 0.9–1.0.

## Results

### Time-Trial

Section and total time for all time-trials are shown in Table 1. There was no Rifle x Sex interaction [*F*_(1, 26)_ = 2.593, *p* = 0.119] for time-trial performance. However, there was a main effect for both Rifle [*F*_(1, 26)_ = 86.829, *p* < 0.001] and Sex [*F*_(1, 26)_ = 49.113, *p* < 0.001]. Time-trials were faster for males compared with females (mean difference: 97 ± 73 s) and No-Rifle compared to With-Rifle (mean difference: 16 ± 9 s).

**Table 1:** Section and total time (s) for both sexes with and without rifle carriage.

**Section**	**Female**		**Male**	
	**With-Rifle**	**No-Rifle**	**With-Rifle**	**No-Rifle**
F1	36 ± 5	36 ± 5	29 ± 2	28 ± 2
UH1	27 ± 4	25 ± 5	18 ± 2	17 ± 3
DH1	13 ± 2	13 ± 2	11 ± 2	12 ± 1
UH2	56 ± 8	54 ± 10	40 ± 4	38 ± 5
DH2	8 ± 1	9 ± 1	8 ± 1	8 ± 1
UH3	45 ± 6	43 ± 6	33 ± 3	31 ± 3
DH3	24 ± 3	24 ± 3	21 ± 4	23 ± 4
UH4	67 ± 13	62 ± 12	47 ± 8	42 ± 7
DH4	15 ± 3	14 ± 1	14 ± 2	13 ± 2
UH5	47 ± 6	43 ± 5	34 ± 4	33 ± 4
F2	21 ± 3	19 ± 1	16 ± 2	16 ± 2
DH5	28 ± 2	27 ± 2	26 ± 2	26 ± 2
UH6	25 ± 5	24 ± 4	20 ± 2	19 ± 3
DH6	15 ± 1	14 ± 1	14 ± 1	14 ± 1
UH7	16 ± 3	15 ± 3	12 ± 1	11 ± 1
DH7	12 ± 1	12 ± 2	11 ± 4	12 ± 3
F3	34 ± 5	33 ± 7	31 ± 6	30 ± 7
TOTAL^*^^#^	488 ± 51	469 ± 49	388 ± 21	375 ± 25

*All values are mean ± SD*;

* *indicates significant main effect for Sex (p <0.05)*;

# *indicates main effect for Rifle (p <0.05). UH, Uphill; F, Flat; DH, Downhill*.

### Physiological Data

**Figure 3:**
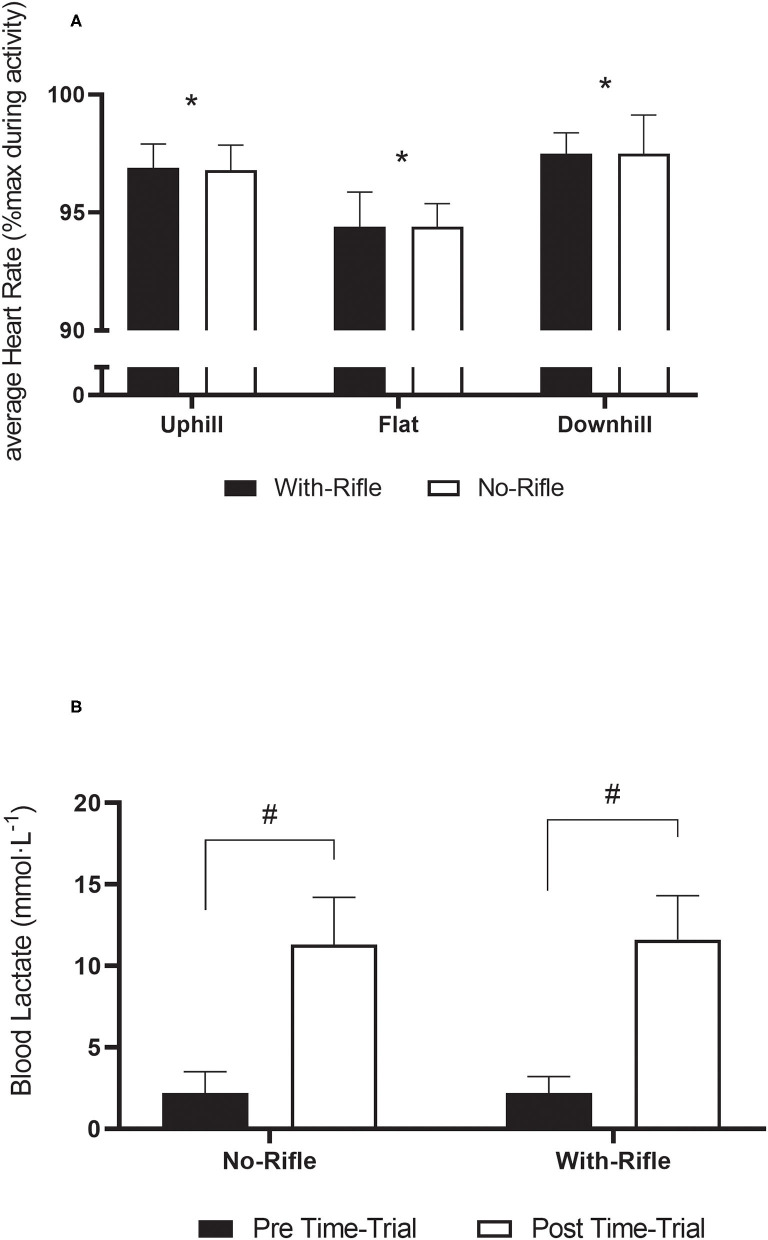
Heart rate **(A)** and blood lactate **(B)** responses to the time-trials. Mean ± standard deviation. *Indicates different to With-Rifle (*p* < 0.001); ^#^indicates different to pre time-trial (*p* < 0.001).

Physiological data (HR and Bla) are displayed in Figure 3. For HR, there was no Gradient x Rifle interaction [*F*_(2, 54)_ = 0.023, *p* = 0.914] or main effect for Rifle [*F*_(1, 26)_ = 0.899, *p* = 0.352]. However, there was a main effect for Gradient [*F*_(2, 54)_ = 225.442, *p* < 0.001]. All pairwise comparisons between Gradient were different (*p* < 0.001). HR was greatest during Downhill sections (98 ± 1%), followed by Uphill (97 ± 1%) and was lowest in the Flat sections (94 ± 2%).

For Bla, there was no Rifle x Timing interaction [*F*_(1, 28)_ = 0.393, *p* = 0.521]. Also, there was no main effect for Rifle [*F*_(1, 28)_ = 0.290, *p* = 0.595], suggesting that rifle carriage has no impact on Bla and that the rest periods between time-trials was sufficient for recovery. As expected there was a timing effect [*F*_(1, 28)_ = 542.338, *p* < 0.001], where Bla was elevated post time-trial (11.4 ± 2.8 mmol·L^−1^) compared to pre time-trial (2.2 ± 1.1 mmol·L^−1^).

### PlayerLoad™

**Figure 4:**
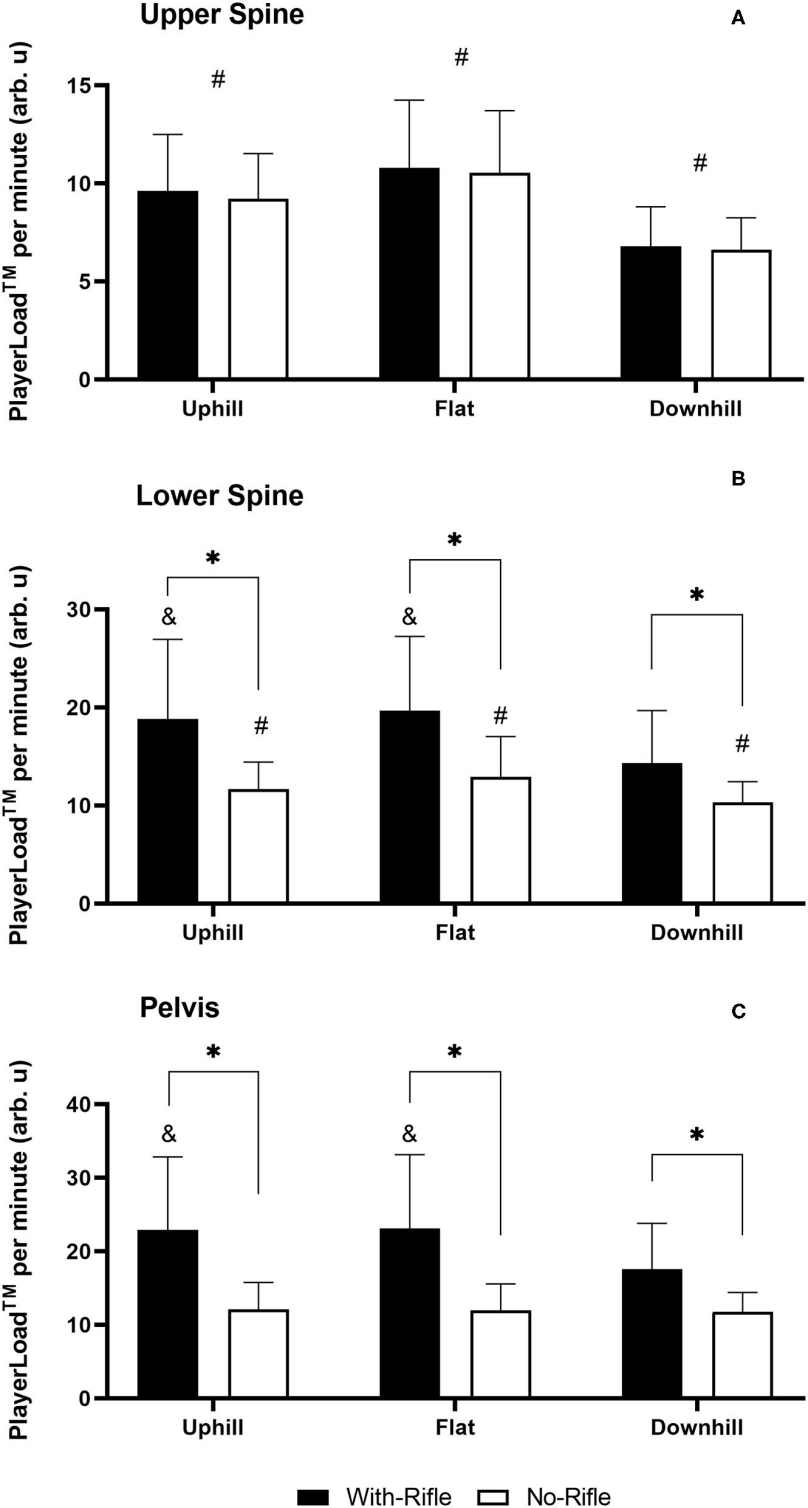
PlayerLoad™ per minute With-Rifle and No-Rifle for all gradients for each sensor position. **(A)** Upper Spine; **(B)** Lower Spine; and **(C)** Pelvis. Mean ± standard deviation. *different to With-Rifle within condition (*p* < 0.001); ^#^different to all other gradients (*p* < 0.001); ^&^different to Downhill within condition (*p* < 0.001).

Figure 4 displays the mean PL·min^−1^ across all conditions. There was a 3-way Rifle x Gradient x Sensor Position interaction [*F*_(4, 104)_ = 10.641, *p* < 0.001]. At the Upper-Spine there was a main effect for Gradient [*F*_(2, 54)_ = 85.590, *p* < 0.001] where all gradients were statistically different (*p* < 0.001). PL·min^−1^ was greatest during Flat sections (10.7 ± 3.3 arb.u), followed by Uphill (9.4 ± 2.6 arb.u) and was lowest for Downhill sections (6.7 ± 1.8 arb.u). At the Lower-Spine there was a Rifle x Gradient interaction [*F*_(2, 52)_ = 30.944, *p* < 0.001]. PL·min^−1^ was greater With-Rifle compared to No-Rifle during all Gradients (*p* < 0.001 for all). With-Rifle, PL·min^−1^ was greater for Uphill and Flat sections compared to Downhill (*p* < 0.001 for both), but there was no difference between Uphill and Flat sections (*p* = 0.556). However, for No-Rifle, PL·min^−1^ was different between all Gradients (*p* < 0.001 for all), being greatest for Flat sections (12.9 ± 4.1 arb. u), followed by Uphill (11.7 ± 2.7 arb. u) and was lowest for Downhill (10.3 ± 2.1 arb. u). At the Pelvis there was a Rifle x Gradient interaction [*F*_(2, 52)_ = 24.046, *p* < 0.001]. PL·min^−1^ was greater With-Rifle compared to No-Rifle during all Gradients (*p* < 0.001 for all). With-Rifle, PL·min^−1^ was greater for Uphill and Flat sections compared to Downhill (*p* < 0.001 for both), but no difference between Uphill and Flat sections (*p* = 1.000). For No-Rifle, PL·min^−1^ was similar across all Gradients (*p* = 1.000 for all).

### Average Net Force

**Figure 5:**
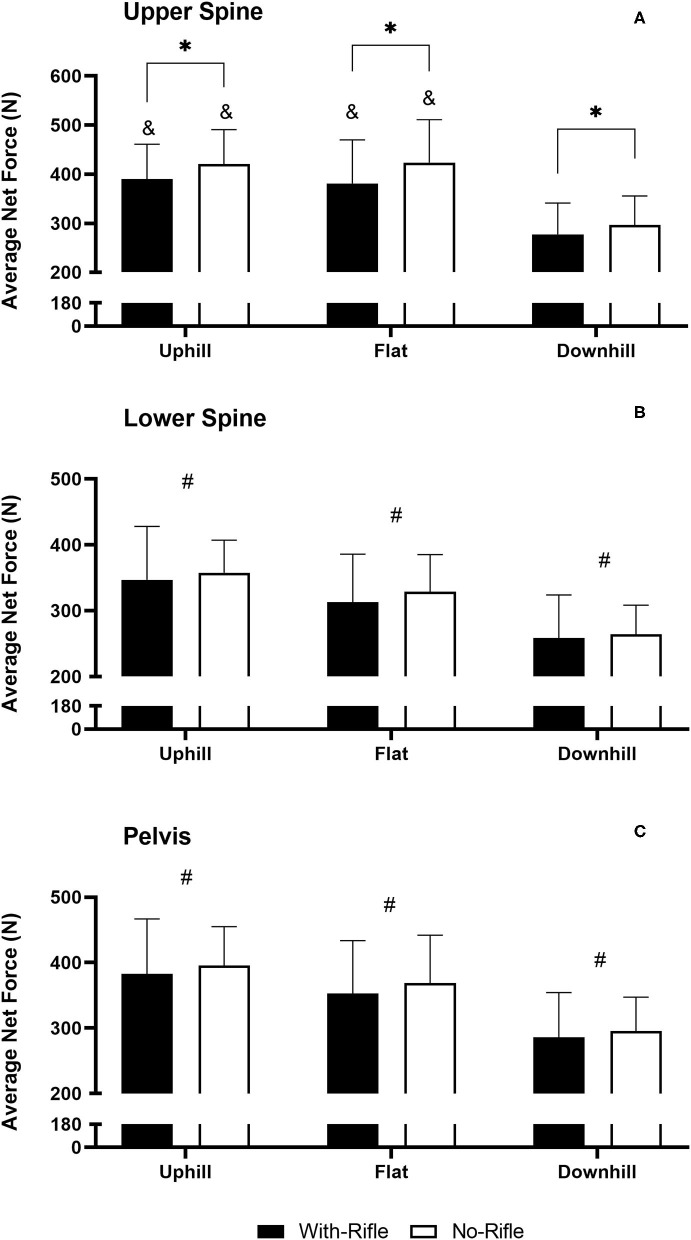
Average Net Force With-Rifle and No-Rifle for all gradients for each sensor position. **(A)** Upper Spine; **(B)** Lower Spine; and **(C)** Pelvis. Mean ± standard deviation. *different to With-Rifle within condition (*p* ≤ 0.003); ^#^different to all other gradients (*p* < 0.001); ^&^different to Downhill within condition (*p* < 0.001).

Figure 5 displays the AvF_Net_ across all conditions. There was a 3-way Rifle x Gradient x Sensor Position interaction [*F*_(4, 104)_ = 2.921, *p* = 0.037]. At the Upper-Spine there was a Rifle x Gradient interaction [*F*_(2, 54)_ = 8.494 *p* < 0.001]. AvF_Net_ was greater for No-Rifle compared to With-Rifle during Uphill (*p* < 0.001), Downhill (*p* = 0.003) and Flat (*p* < 0.001) Gradients. With-Rifle, AvF_Net_ was greater for Uphill and Flat sections compared to Downhill (*p* < 0.001 for both). There was no difference between Uphill and Flat sections (*p* = 0.632). For No-Rifle, AvF_Net_ was greater for Uphill and Flat sections compared to Downhill (*p* < 0.001 for both). There was no difference between Uphill and Flat sections (*p* = 1.000). At the Lower-Spine there was a Gradient effect [*F*_(2, 54)_ = 203.904, *p* < 0.001]. All pairwise comparisons for Gradient were different (*p* < 0.001 for all). AvF_Net_ was greatest during Uphill (352 ± 67 N), followed by the Flat sections (321 ± 65 N), and with Downhill sections the lowest (261 ± 56 N). At the Pelvis there was a Gradient effect [*F*_(2, 54)_ = 118.983, *p* < 0.001]. All pairwise comparisons for Gradient were different (*p* < 0.001 for all). AvF_Net_ followed the expected response where the Uphill section was the greatest (389 ± 73 N), followed by the Flat sections (361 ± 77 N), with Downhill the lowest (291 ± 60 N).

### Correlations

**Figure 6:**
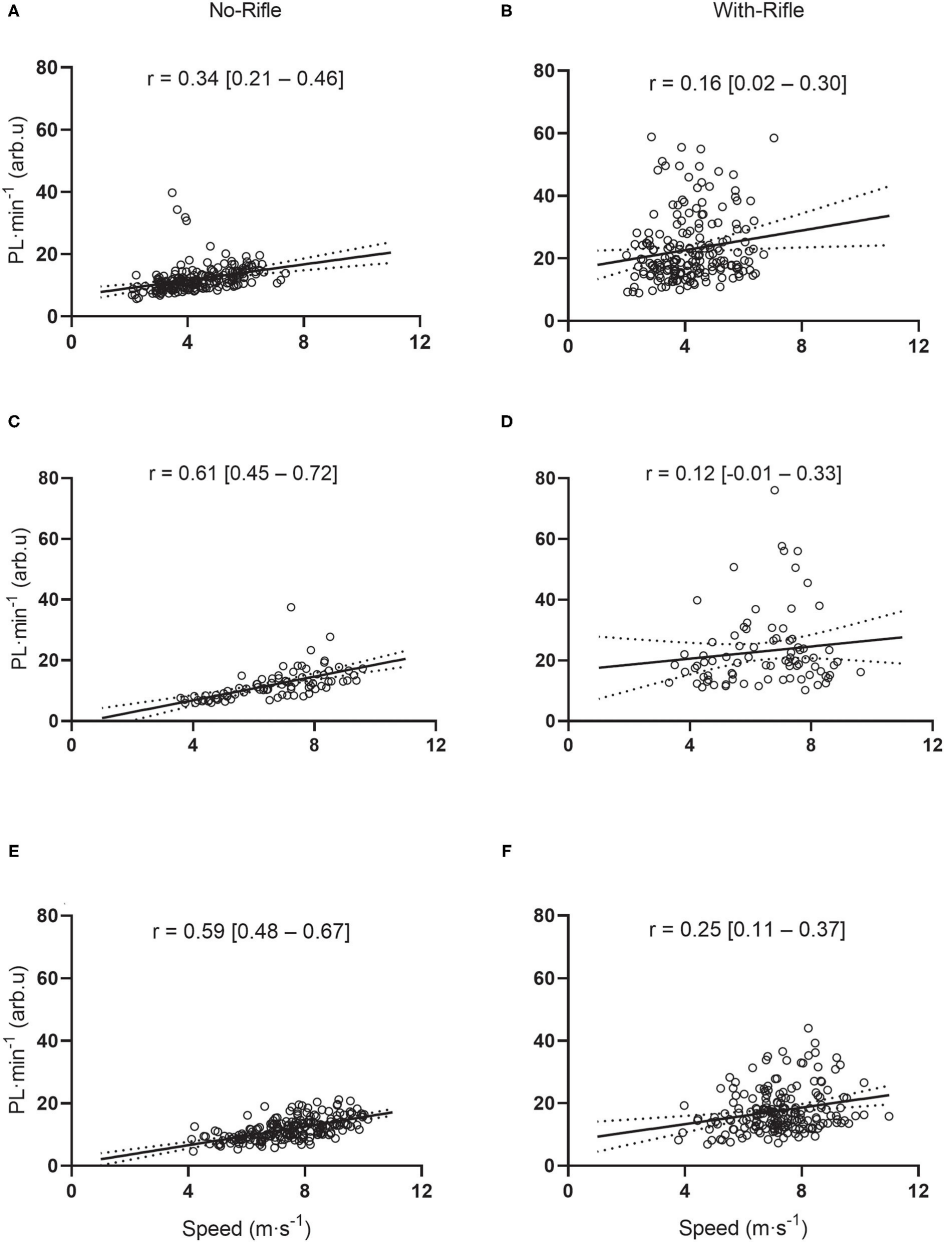
Scatterplots displaying relationships between PlayerLoad™ per minute and skiing speed during Uphill, Flat and Downhill gradients with and without the biathlon rifle. Each dot represents an individual speed (x) vs. PlayerLoad™ per minute (y) (for the pelvis sensor) for every Uphill **(A,B)**, Flat **(C,D)** and Downhill **(E,F)** section. Panels on the left are for No-Rifle; Panels on the right are for With-Rifle. Pearson correlation coefficient (*r*) with 95% confidence interval shown.

Figure 6 displays the correlations between PL·min^−1^ (for the pelvis sensor) and skiing speed for Uphill (Figures 6A,B), Flat (Figures 6C,D) and Downhill (Figures 6E,F) track sections for both No-Rifle and With-Rifle conditions. For Uphill sections, the correlations between skiing speed and PL·min^−1^ were small for With-Rifle [*r* = 0.16 (95%CI: 0.02–0.30), *p* = 0.024] and moderate for No-Rifle [*r* = 0.34 (95%CI: 0.21–0.46), *p* < 0.001; Figures 6A,B]. For Flat sections, the correlations between skiing speed and PL·min^−1^ were insignificant and small for With-Rifle [*r* = 0.12 (95%CI: −0.01 to 0.33), *p* = 0.315] and large for No-Rifle [*r* = 0.61 (95%CI: 0.45–0.72), *p* < 0.001; Figures 6C,D]. For Downhill sections, the correlations between skiing speed and PL·min^−1^ were small for With-Rifle [*r* = 0.25 (95%CI: 0.11–0.37), *p* < 0.001] and large for No-Rifle [*r* = 0.59 (95%CI: 0.48–0.67), *p* < 0.001; Figures 6E,F].

**Figure 7:**
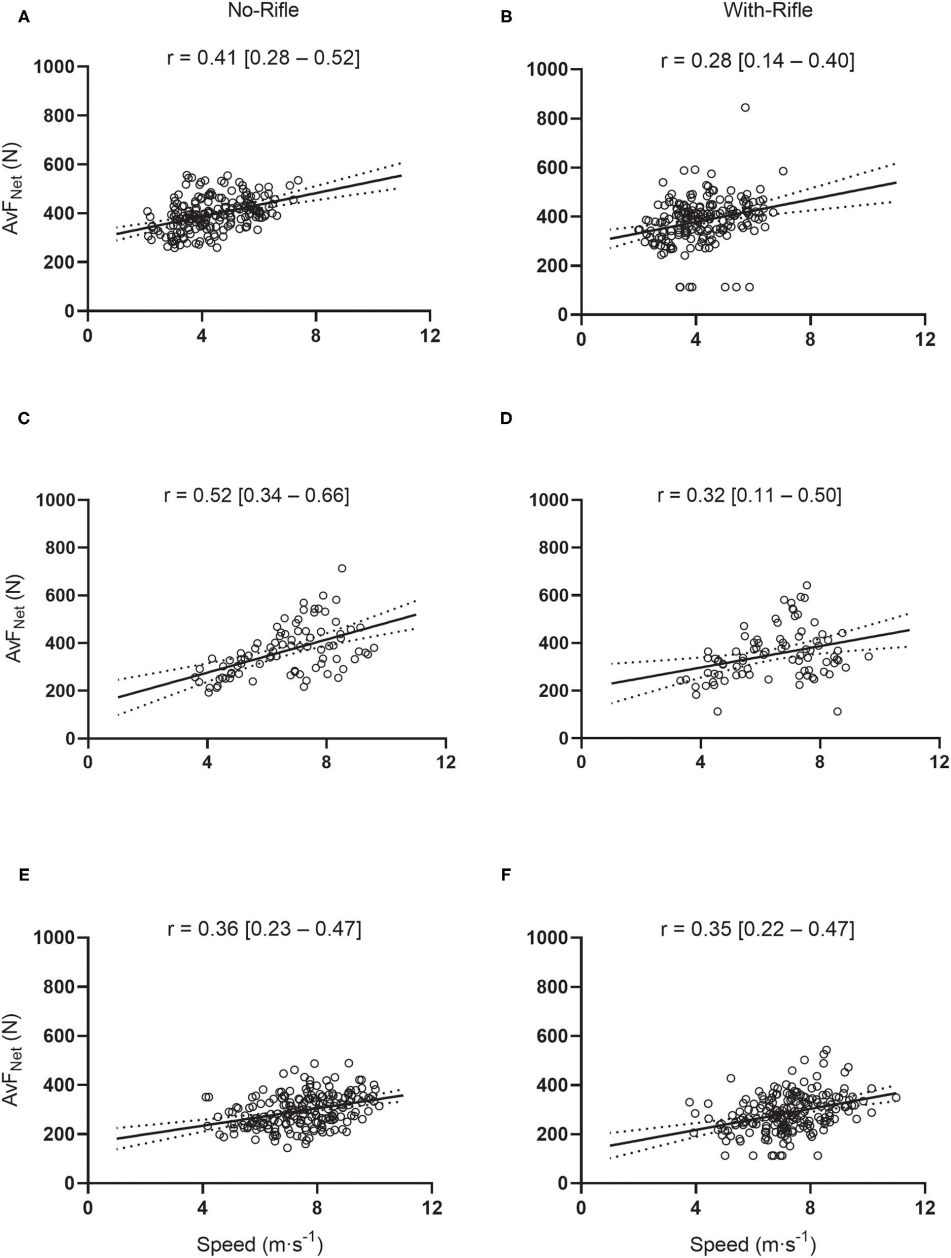
Scatterplots displaying relationships between Average Net Force and skiing speed during Uphill, Flat and Downhill gradients with and without the biathlon rifle. Each dot represents an individual speed (x) vs. Average Net Force (y) (for the pelvis sensor) for every Uphill **(A,B)**, Flat **(C,D)** and Downhill **(E,F)** section. Panels on the left are for No-Rifle; Panels on the right are for With-Rifle. Pearson correlation coefficient (*r*) with 95% confidence interval shown.

Figure 7 displays the correlations between AvF_Net_ (for the pelvis sensor) and skiing speed for Uphill (Figures 7A,B), Flat (Figures 7C,D) and Downhill (Figures 7E,F) track sections for both No-Rifle and With-Rifle conditions. For Uphill sections, the correlations between skiing speed and AvF_Net_ were small for With-Rifle [*r* = 0.28 (95%CI: 0.14–0.40), *p* < 0.001] and moderate for No-Rifle [*r* = 0.41 (95%CI: 0.28–0.52), *p* < 0.001; Figures 7A,B]. For Flat sections, the correlations between skiing speed and AvF_Net_ were moderate for With-Rifle [*r* = 0.32 (95%CI: 0.11–0.50), *p* = 0.003] and moderate for No-Rifle [*r* = 0.52 (95%CI: 0.34–0.66), *p* < 0.001; Figures 7C,D]. For Downhill sections, the correlations between skiing speed and AvF_Net_ were moderate for With-Rifle [*r* = 0.35 (95%CI: 0.22–0.47), *p* < 0.001] and moderate for No-Rifle [*r* = 0.36 (95%CI: 0.23–0.47), *p* < 0.001; Figures 7E,F].

## Discussion

This is the first study to quantify accelerometry-derived measures of external demand, as well as physiological responses to a XC skiing time-trial, performed in ecologically valid on-snow conditions, with and without the biathlon rifle. The main findings of this study were: (1) the biathlon rifle had little impact on the mean physiological responses, as neither HR nor Bla were elevated as a result of rifle carriage. However, the rifle impacted skiing time-trial performance, indicating the rifle impacted relative physiological intensity; (2) the magnitude of accelerometry-derived measures followed the expected response of exercise intensity during biathlon skiing; (3) significant positive linear relationships were identified between skiing speed and accelerometer-derived metrics during Uphill, Flat and Downhill skiing.

This study demonstrated that mean HR and Bla was not impacted by rifle carriage. However, it is not surprising that mean HR and Bla responses were not different given both time-trials (with and without rifle) were completed at maximum effort. Because rifle carriage significantly impacted the overall skiing time-trial performance in the present study, it can be concluded that rifle carriage did impact the relative physiological intensity. These findings confirm previous research where it was observed that rifle carriage increases metabolic work, HR and Bla during roller-skiing on a treadmill (Rundell and Szmedra, 1998; Stöggl et al., 2015; Jonsson Kårström et al., 2019). Further, the results of the present study extends previous research by demonstrating the physiological responses to biathlon skiing in a more ecologically valid, on-snow, setting.

This study identified that HR was greatest during the downhill sections of the skiing course and not during the flat or uphill sections. Similar research has also previously found average HR values to be the greatest during downhill sections of a skiing course (Bilodeau et al., 1991). This result might be explained by the methodological approach of averaging HR during each course gradient. In this particular course profile, downhill sections were preceded by an uphill section in nearly all cases. It is possible that other patterns could be observed if the course profile was different. Further, it is also possible that greater HR during downhill sections can be explained by the delay associated with cardiorespiratory acceleration and decelerations (Xu and Rhodes, 1999). It is common in biathlon and XC skiing for athletes to complete a high-intensity effort over the summit of each climb in order to maximize their momentum for the following downhill section (Ihalainen et al., 2022). This means that the HR response during the downhill sections might be reflecting the greater intensity during the preceding moments of high-intensity effort over the summit of each climb. Despite this potential limitation, HR is used almost exclusively as an indicator of exercise intensity during biathlon skiing (Kårström et al., 2021; Talsnes et al., 2021). It is acknowledged that measurement of the average exercise intensity from HR might still be useful because overestimation and underestimation of intensities over the duration of intermittent exercise might balance. However, it is likely that HR is not reflecting instantaneous exercise intensity, which might be important for an athlete to monitor within a training session or a race. Accordingly, novel methods of monitoring exercise intensity in skiing are warranted.

The magnitude of the accelerometry-derived metrics was typically greatest during uphill and flat sections and were lowest during downhill sections. This response likely resembles the actual external demands of the activity. Considering that the accelerometry-derived metrics represent dynamic bodily movements and not physiological demands, these results demonstrate that the bodily impacts and movement are still quite high during downhill sections. This is not surprising given downhill sections are associated with high speed turns and still require considerable expression of force to maintain body position, stability and velocity throughout a turn (Supej et al., 2011; Bucher Sandbakk et al., 2014; Sandbakk et al., 2014). Nevertheless, bodily movements and impacts remained greatest during uphill and flat sections where biathletes utilize different “gears” of skiing in order to best maintain a high speed over the duration of the course (Andersson et al., 2010). Utilization of these “gears” requires considerable use of dynamic bodily movements and hence high accelerometry-derived responses.

It is important to consider that this is the first study to use accelerometery-derived metrics to monitor the external demands in skiing. Currently, the only method of monitoring external intensity in skiing is through measuring skiing speed. It is acknowledged that locomotion speed is not necessarily the strongest indictor of external exercise intensity during skiing because different snow and weather conditions can greatly impact the skiing speed. Regardless, it is currently the only available measure of external exercise intensity practically available to skiers and coaches.

Significant positive linear relationships were identified between skiing speed and accelerometer-derived metrics during nearly all skiing sections. The one exception was an insignificant, small positive correction between PL·min^−1^ and skiing speed during Flat sections With-Rifle. It is logical that there are positive associations between force (accelerations) and skiing speed during uphill sections because skiers utilize the aforementioned “gears” in order to generate propulsive forces to move themselves uphill against gravity (Gløersen et al., 2018; Swarén and Eriksson, 2019). Additionally, it is logical that high speeds during downhill and flat track sections are also associated with higher forces. As previously discussed, although downhill sections might be associated with little to no metabolic work, these sections are still associated with considerable expression of force (Supej et al., 2011; Bucher Sandbakk et al., 2014; Sandbakk et al., 2014). Interestingly, correlations between accelerometry-derived metrics and skiing speed were consistently stronger for No-Rifle compared to With-Rifle, particularly for PL·min^−1^. This might be explained be the fact that at the pelvis location (which is the location the correlations are derived from) PL·min^−1^ was significantly greater with With-Rifle compared to No-Rifle (Figure 4C). In addition, the With-Rifle condition resulted in slower times (i.e., slower speeds) (Table 1). On the other hand, AvF_Net_ was more consistent across the With-Rifle and No-Rifle conditions.

This study has presented the utility of accelerometer-derived metrics during ecologically valid settings. Further investigation of the internal validity of these metrics is warranted by investigating during laboratory-controlled conditions, where accelerometry-derived metrics can be compared to measures of power output and internal physiological responses, such as $\overset{∙}{\text{V}}{\text{O}}_{2}$ and energy expenditure during standardized conditions. In particular, AvF_Net_ recorded at the Lower-Spine or Pelvis (i.e., sensor locations close to the center of mass) appears to be the most promising metric for measuring the external demand in XC skiing and biathlon. This is because the response at these two sensor locations followed the expected response, being greatest for Uphill, then Flat and lowest for Downhill sections. Additionally, rifle carriage had little impact on the magnitude of the AvF_Net_ response at these sensor positions, which also aligns with the physiological responses demonstrated in the present study. Further, the relationships between AvF_Net_ and skiing speed were far more consistent between With-Rifle and No-Rifle in comparison to PL·min^−1^. On the hand, it seems clear that PL·min^−1^ with sensor positions at the Lower-Spine and Pelvis, as well as AvF_Net_ recorded at the Upper-Spine are not suitable to determine the exercise intensity in biathlon. At these sensor locations there was a difference in PL between With-Rifle and No-Rifle conditions.

## Practical Applications

This research provides XC skiers, biathletes and their coaches with valuable information pertaining to the utility of physiological and accelerometry-derived metrics for monitoring exercise demands. Firstly, coaches and athletes need to recognize that HR does not necessarily reflect instantaneous exercise intensity, so caution must be used when interpreting these data at any given point in time. Regardless, HR might still provide useful information regarding average exercise intensity over the duration of a race or training session. Accelerometry-derived metrics might provide a method with utility for monitoring the external demands during biathlon and XC skiing. In particular, AvF_Net_ with sensors located close to the center of mass displayed greatest utility because it followed the expected response of external intensity where responses were greatest during uphill sections, followed by flats and lowest during downhills. In addition, there were significant positive relationships between AvF_Net_ and skiing speed ranging from small to large. AvF_Net_ has potential to be used in order to monitor the external demands during training and competition, as well as for the design of training programs to replicate competition intensities. However, further examinations are required before these metrics can be recommended for use in practice.

## Conclusions

The biathlon rifle impacted skiing performance and the relative physiological intensity. In the present study HR was greatest during the downhill sections of the skiing course, which likely reflects the greater effort imposed during the preceding uphill. The accelerometry-derived approach used in this study provides the potential of a novel method of monitoring the external demands during skiing. In particular, AvF_Net_ recorded at the Lower-Spine or Pelvis (i.e., sensor locations close to the center of mass) appears to be the most promising metric for measuring the external demand in biathlon. Further research is required before AvF_Net_ can be recommended for use in practice.

## Data Availability Statement

The raw data supporting the conclusions of this article will be made available by the authors, without undue reservation.

## Ethics Statement

The studies involving human participants were reviewed and approved by the Regional Ethical Board in Umeå, Sweden (#2016-506-31M). The patients/participants provided their written informed consent to participate in this study.

## Author Contributions

CS participated in the design of the study, contributed to data collection and was responsible for data analysis, statistical analysis, and writing the manuscript. LS and MB participated in the design of the study and were responsible for data collection. MJ, ML, and GB participated in the design of the study and provided editorial assistance in manuscript preparation. All authors have read and approved the final version of the manuscript.

## Funding

This study was financed by the World Championships Region, Östersund. The results of the current study do not constitute endorsement of any commercial products by the authors or the journal.

## Conflict of Interest

The authors declare that the research was conducted in the absence of any commercial or financial relationships that could be construed as a potential conflictof interest.

## Publisher's Note

All claims expressed in this article are solely those of the authors and do not necessarily represent those of their affiliated organizations, or those of the publisher, the editors and the reviewers. Any product that may be evaluated in this article, or claim that may be made by its manufacturer, is not guaranteed or endorsed by the publisher.
